# Vertebrae-Based Body Length Estimation in Crocodylians and Its Implication for Sexual Maturity and the Maximum Sizes

**DOI:** 10.1093/iob/obaa042

**Published:** 2020-11-24

**Authors:** Masaya Iijima, Tai Kubo

**Affiliations:** 1 Department of Biological Sciences, Clemson University, Clemson, SC 29634, USA; 2 Nagoya University Museum, Furocho, Chikusa-Ku, Nagoya, Aichi 464-8601, Japan; 3 Engineering Research Center for Mineral Resources and Mine Environments, School of Resource and Environmental Engineering, Hefei University of Technology, 193 Tunxi Road, Baohe, Hefei, Anhui 230009, China; 4 The University Museum, The University of Tokyo, 7-3-1 Hongo, Bunkyo-ku, Tokyo 113-0033, Japan

## Abstract

Body size is fundamental to the physiology and ecology of organisms. Crocodyliforms are no exception, and several methods have been developed to estimate their absolute body sizes from bone measurements. However, species-specific sizes, such as sexually mature sizes and the maximum sizes were not taken into account due to the challenging maturity assessment of osteological specimens. Here, we provide a vertebrae-based method to estimate absolute and species-specific body lengths in crocodylians. Lengths of cervical to anterior caudal centra were measured and relations between the body lengths (snout–vent and total lengths [TLs]) and lengths of either a single centrum or a series of centra were modeled for extant species. Additionally, states of neurocentral (NC) suture closure were recorded for the maturity assessment. Comparisons of TLs and timings of NC suture closure showed that most extant crocodylians reach sexual maturity before closure of precaudal NC sutures. Centrum lengths (CLs) of the smallest individuals with closed precaudal NC sutures within species were correlated with the species maximum TLs in extant taxa; therefore, the upper or lower limit of the species maximum sizes can be determined from CLs and states of NC suture closure. The application of the current method to noncrocodylian crocodyliforms requires similar numbers of precaudal vertebrae, body proportions, and timings of NC suture closure as compared to extant crocodylians.

## Introduction

Body size is an important trait related to most biological aspects of organisms (McMahon and Bonner 1983; Peters 1983; Schmidt-Nielsen 1984). In extant crocodylians, body size is associated with per-mass bite force (Erickson et al. 2003, 2014), terrestrial and aquatic locomotor capability (Bustard and Singh 1977a; Seebacher et al. 2003; Hutchinson et al. 2019), diving behavior (Cott 1961; Campbell et al. 2010; Grigg and Kirshner 2015), thermal relations (Grigg et al. 1998; Seebacher et al. 1999), movements and dispersal patterns (Hutton 1989; Kay 2004), and reproductive characteristics (e.g., egg mass and clutch size: Thorbjarnarson 1996; Verdade 2001). Differences in adult sizes facilitate niche segregation in sympatric crocodylian species (Ouboter 1996) and may result in differential interaction with the prey population and the surrounding environment (Somaweera et al. 2020). In the geologic time scale, macroevolutionary analysis of body size in extinct crocodyliforms can illuminate the rate and mode of the evolution and factors responsible for the long-term disparity patterns (Godoy et al. 2019; Gearty and Payne 2020; Solórzano et al. 2020).

Several osteological proxies have been used to estimate body size (snout–vent length [SVL], TL, and body mass) of crocodyliforms and their relatives: cranial measurements (e.g., head length and width: Wermuth 1964; Greer 1974; Webb and Messel 1978; Hutton 1987; Hall and Portier 1994; Woodward et al. 1995; Verdade 2000; Sereno et al. 2001; Hurlburt et al. 2003; Wu et al. 2006; Whitaker and Whitaker 2008; Young et al. 2011, 2016; Fukuda et al. 2013; Scheyer et al. 2013; O’Brien et al. 2019); appendicular bone measurements (e.g., femur length and width: Brochu 1992; Farlow et al. 2005; Young et al. 2011, 2016); scute length (Bustard and Singh 1977b). Recently, O’Brien et al. (2019) proposed a phylogenetic prediction of body size from the head width, which will be a strong tool when the animal’s phylogenetic position is known within Crocodylia.

All previous methods estimated absolute body sizes of crocodyliforms, while species-specific sizes (e.g., sexually mature sizes and the maximum sizes) were not considered due to the lack of reliable maturity indicator in the skull and the appendicular skeleton, except for the histological features (Erickson and Brochu 1999; Lee 2004; Woodward et al. 2011). Maturity of the skull has been assessed using the external morphological characteristics including proportional relations of the skull regions, the shape of supratemporal fenestrae, and the degree of toothrow festooning (Mook 1921a; Kälin 1933; Iordansky 1973; Dodson 1975). However, these characteristics are often size-dependent instead of maturity-dependent (Iijima 2017) and can at best estimate broad ontogenetic stages (e.g., juvenile and adult). Unlike most tetrapods, skull sutures remain open throughout life in crocodylians, thus the degree of skull suture closure cannot be used for the maturity assessment (Bailleul et al. 2016). Osteological maturity can be assessed by the presence/absence of scars and tuberosity in the appendicular skeleton, though the applicability of this method to fossil forms is untested (Brochu 1992, 1996). The long bone surface texture has no relation to the skeletal maturity in crocodylians (Tumarkin-Deratzian et al. 2007). Osteological correlates of sexual maturity are only known in the snouts of male *Gavialis gangeticus* (Martin and Bellairs 1977; Hone et al. 2020), and few extinct taxa are expected to yield those.

The most popular and noninvasive indicator of skeletal maturity in crocodyliforms and their kin is neurocentral (NC) suture closure in vertebrae (Mook 1933a; Brochu 1996; Irmis 2007). In extant crocodylians, the NC suture closes sequentially from distal caudal vertebrae to anterior cervical vertebrae during ontogeny (Brochu 1996; Ikejiri 2012), and dwarf species show closure of precaudal NC sutures at smaller sizes than large species (Brochu 1996). Although suture closure timings may vary interspecifically, the same caudal to cranial closure sequence is likely shared among crocodyliforms (Irmis 2007).

In this study, we focus on crown-group crocodylians and provide a method to estimate absolute and species-specific body lengths of extinct crocodylians using the centrum lengths (CLs) and states of NC suture closure. Crown-group crocodylians share the same number of precaudal vertebrae (9 cervical, 15 dorsal, and 2 sacral vertebrae: Reese 1915; Mook 1921b; Hoffstetter and Gasc 1969; Iijima and Kubo 2019), allowing the body size estimation based on incompletely preserved precaudal vertebrae. Recently, sacralization of the last dorsal vertebra was reported in a Miocene caimanine *Purussaurus mirandai* (Scheyer et al. 2019), although it would not change the total number of precaudal vertebrae. Indeed, bilateral sacralization of the last dorsal or the first caudal vertebra similar to that in *P. mirandai* was also found in few extant crocodylian species (*Osteolaemus tetraspis* [AMNH 69057]; *G. gangeticus* [FMNH 98864]: M.I. personal observation). Here, we first model relations of the absolute SVL and TL against the lengths of either a single centrum or a series of centra in extant crocodylians. We then establish the relations of sexually mature body lengths and the species maximum body lengths against timings of NC suture closure in extant crocodylians. Finally, we estimate absolute and the species maximum body lengths of extinct taxa using the models of extant crocodylians.

## Materials and methods

Institutional abbreviations: AMNH, American Museum of Natural History, New York, NY, USA; CM, Carnegie Museum of Natural History, Pittsburgh, PA, USA; GMNH, Gunma Museum of Natural History, Tomioka, Gunma, Japan; MOU, Museum of Osaka University, Toyonaka, Osaka, Japan; MZKB, Zaykaber Museum, Yangon, Myanmar; NMNS, National Museum of Natural Science, Taichung, Taiwan; NTM, Museum and Art Gallery of the Northern Territory, Darwin, Australia; QM, Queensland Museum, Brisbane, Australia; TMM, Texas Memorial Museum, Austin, TX, USA; UF, Florida Museum of Natural History, Gainesville, FL, USA; USNM, Smithsonian National Museum of Natural History, Washington DC, USA; YPM, Peabody Museum of Natural History, Yale University, New Haven, CT, USA.

### Specimens, measurements, and assessments of NC suture closure

**Fig. 1:**
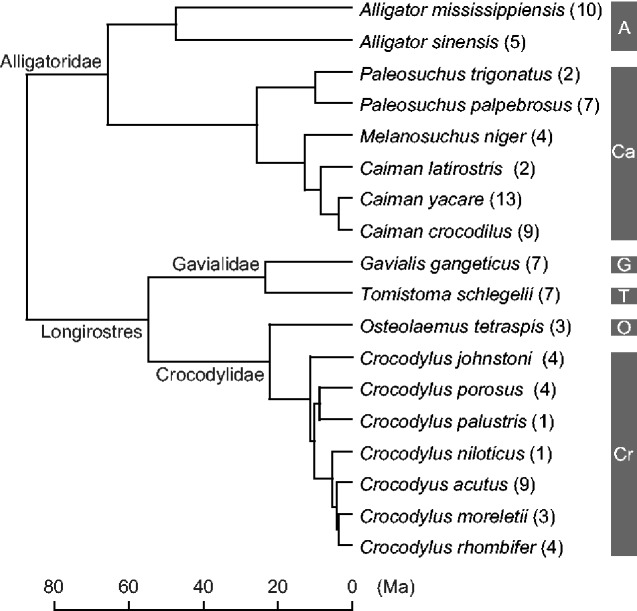
(A) Time-calibrated tree of 18 extant crocodylians examined in this study. The tree topology, root age (87.14 Myr), and divergence times were adopted from Oaks’ (2011) analysis with 90 Myr upper limit on the root age. Numbers in parentheses are sample sizes for each species. Subfamily: A, Alligatorinae; Ca, Caimaninae; Cr, Crocodylinae; G, Gavialinae; O, Osteolaeminae; T, Tomistominae.

For extant crocodylians, axial skeletons of 95 individuals from 18 species were sampled (Fig. 1; Supplementary data, Table S1). Our sampling covered all six extant subfamilies of crocodylians (Alligatorinae, Caimaninae, Crocodylinae, Osteolaeminae, Tomistominae, and Gavialinae: Brochu 2003). Sixty-seven individuals were wild-caught and 28 individuals were captive-raised or without locality data. The sample was variously represented by juveniles to adults. Sexes were known in few individuals, thus we pooled males, females, and unknown sex for the following analyses. The specimens were primarily dry cervical to anterior caudal vertebrae that allow CL measurements and assessments of states of NC suture closure.

**Fig. 2:**
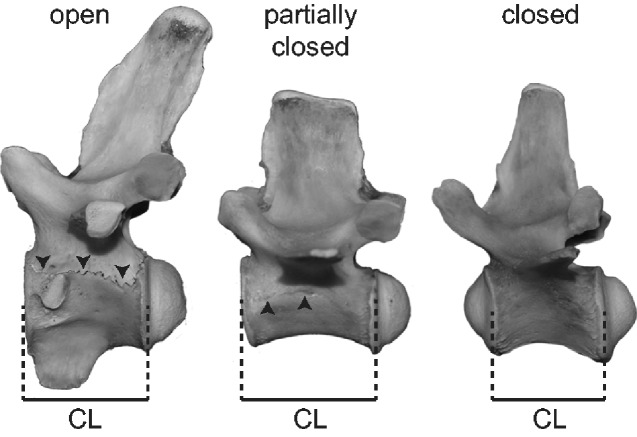
Measurements of CLs and the assessment of NC suture closure. From left to right, 1st dorsal, 12th dorsal, and 1st caudal vertebrae of *C. johnstoni* (QM J58446). Arrows represent open sutures.

CLs of the axis to 10th caudal vertebra were measured with the Tresna digital caliper to the nearest 0.01 mm. A CL was defined as the craniocaudal length along the mid-height of the centrum excluding the condyle (convex articular surface) (Iijima and Kubo 2019; Fig. 2). The axis CL includes the odontoid process, and the biconvex first caudal CL excludes both anterior and posterior condyles. Among 95 individuals examined, 46 individuals preserved a complete series of cervical to anterior caudal vertebrae, while 49 individuals were missing at least one centrum. A missing CL was estimated using the equation CL_av_(CL_sum.ind_/CL_sum.av_), where CL_av_ is the average CL for the missing vertebral position in the same species (if not available, the same genus) with complete vertebral series, CL_sum.ind_ is the sum of the available CLs in the individual with missing vertebrae, and CL_sum.av_ is the sum of the average CLs of corresponding vertebral positions in the same species (if not available, the same genus).

States of NC suture closure were assessed using the criteria outlined in previous studies (Brochu 1992, 1996; Irmis 2007; Ikejiri 2012; Fig. 2). A fully open suture is the one that is visible along the entire neural arch-centrum contact. Partially closed and fully closed sutures exhibit partly obscured and completely obscured suture lines, respectively. The assessment was solely based on the observation of the external surface of the vertebrae. The degree of internal ossification (Ikejiri 2012) was disregarded here.

### Relations of absolute body lengths (SVLs and TLs) and CLs

SVLs and/or TLs were available in 30 individuals from 10 species. If either SVL or TL was missing in these individuals, SVL–TL conversions were used to calculate the missing value based on the SVL and TL measurements and the conversion equations in the literature (Supplementary data, Table S1).

**Fig. 3:**
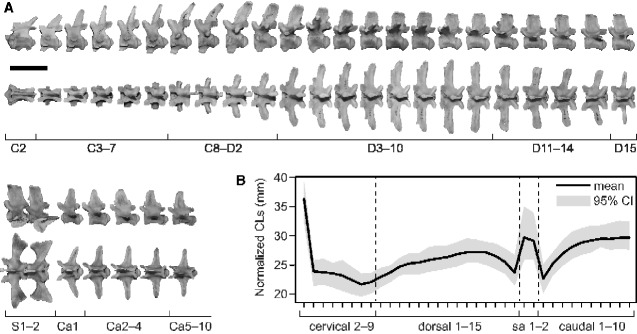
(**a**) Cervical to caudal vertebrae of *C. johnstoni* (QM J58446), illustrating the divisions of vertebrae used in this study. (**B**) CL profile of extant crocodylians. Normalized CLs were calculated as a CL divided by the ratio of the sum of the axis to 10th caudal CLs in the same individual to its average in 95 individuals from 18 species. Scale bar equals 5 cm.

To estimate body lengths in extinct crocodylians, we formulated equations to derive SVLs and TLs from a single vertebra or a series of vertebrae. The axis to 10th caudal vertebra was divided into 10 regions (axis, 3–7th cervical vertebrae, 8th cervical to 2nd dorsal vertebrae, 3–10th, 11–14th, and 15th dorsal vertebrae, 1st and 2nd sacral vertebrae, 1st, 2–4th, and 5–10th caudal vertebrae) (Fig. 3). The regional boundaries of vertebrae were determined by the ease of positional identification and the degree of morphological and CL changes. The relations of SVLs and TLs against the average CLs in each region were modeled for 30 extant individuals with SVL and/or TL records using reduced major axis (RMA) regressions to estimate body lengths from a single vertebra. We also modeled the relations of SVLs and TLs against the sum of CLs in a series of vertebrae (axis to 9th cervical vertebrae, 1–15th dorsal vertebrae, and axis to 15th dorsal vertebrae) with RMA regressions to estimate body lengths of well-preserved fossils. We used RMA instead of ordinary least squares (OLSs) regressions because although OLS is recommended when predicting the depending variable from the independent variable, RMA arguably performs better if a predicted value falls beyond the range of observed values (Smith 2009). Variables were log10 transformed prior to the regressions. RMA regressions were performed with the R package smatr (Warton et al. 2012; R Core Team 2016).

### Relations of species-specific body lengths (sexually mature and the maximum TLs) and timings of NC suture closure

Previous studies of *Alligator mississippiensis* demonstrated that precaudal NC sutures close after both sexes reach sexual maturity, and NC suture closure progresses rapidly toward the axis after the first closure of a sacral centra (Brochu 1996; Ikejiri 2012). Given these observations, we chose the smallest individuals showing partially/fully closed NC sutures in precaudal vertebrae (hereafter referred to as the smallest osteologically mature individuals) in each species for the relative maturity comparison. Species were selected for the analysis if the TL difference between the smallest osteologically mature individual and the nearest sized individual with fully open precaudal vertebrae within species was <30%. Thirteen out of 18 species satisfied the cutoff criteria. Missing SVLs and TLs of the smallest osteologically mature individuals in 13 extant species were calculated using the RMA equations of SVLs and TLs against the sum of the axis to 10th caudal CLs for 30 individuals with the body length records. Male and female sexually mature TLs were qualitatively compared with the smallest osteologically mature TL in each species. We did not conduct statistical tests because sex data were sparse in our sample.

For the species maximum body length estimations, relations between the maximum TLs and the CLs for the smallest osteologically mature individuals in 13 species were modeled using RMA regressions: the average CLs of each vertebral region (axis, 3–7th cervical vertebrae, 8th cervical to 2nd dorsal vertebrae, 3–10th, 11–14th, and 15th dorsal vertebrae, 1st and 2nd sacral vertebrae, 1st, 2–4th, and 5–10th caudal vertebrae) and the sum of CLs in a series of vertebrae (axis to 9th cervical vertebrae, 1–15th dorsal vertebrae, and axis to 15th dorsal vertebrae) were used for the modeling. Variables were log10 transformed prior to the regressions. Both males and females in 13 species were used for the modeling. The analysis of the pooled sex sample might complicate the model interpretation because extant crocodylians are sexually size-dimorphic and the maximum sizes are attained by males (e.g., Webb and Manolis 1989; Vliet 2020). However, males and females reach sexual maturity at similar sizes in most extant species (Ferguson 1985), and the timings of NC suture closure in the pooled sex sample of *A. mississippiensis* are rather uniform (Ikejiri 2012). Therefore, we assumed that suture closure timings relative to the absolute body lengths are similar in males and females in each species. Male and female sexually mature TLs and the maximum TLs in each species were adopted from the literature. SVLs were not used for the species-specific size comparison because TLs were more readily available in larger species. The use of size instead of age as the measure of maturity should be valid because sexual maturity is size-dependent in extant crocodylians (e.g., Webb and Manolis 1989; Vliet 2020). We did not use the phylogenetically generalized least squares model for the species regressions because the phylogenetic signal *λ* (Pagel 1999) was 0 and the null hypothesis of *λ* = 1 was rejected (*P* < 0.001) for all regressions, suggesting a negligible phylogenetic signal. The time-calibrated tree of 13 extant species used for the simultaneous estimations of *λ* (Revell 2010) was based on Oaks’ (2011) tree (Fig. 1), deleting five species that were not included in the analysis. The analysis was performed with the R package caper (Orme et al. 2013).

### Absolute and species-specific body length estimations in extinct crocodyliforms

With the regression models established for extant crocodylians, we estimated the absolute SVLs and TLs and the maximum TLs of extinct crocodylians. The absolute SVLs and TLs were estimated using the relations of SVLs and TLs against the CLs in 30 extant crocodylians with SVL and/or TL records. Similarly, the upper and lower limits of the species maximum TLs were estimated using the relation of the maximum TLs against the CLs of the smallest osteologically mature individuals in 13 extant species. The 95% confidence intervals (CIs) of SVL, TL, and the upper and lower limits of the maximum TL estimates were calculated using 1000 bootstrap replicates of the dataset. Because the smallest osteologically mature individual was defined as the smallest one with partially/fully closed precaudal NC sutures, a specimen with closed precaudal vertebrae can predict the upper limit of the species maximum TL, and a specimen with open caudal vertebrae can predict the lower limit of the species maximum TL. If a species is represented by multiple individuals with closed precaudal vertebrae and open caudal vertebrae, then the maximum TL estimate can be constrained by upper and lower limits. A single vertebra or a series of vertebrae was used for the maximum TL estimation, depending on the preservation of fossils.

We applied the vertebrae-based absolute and maximum body length estimations to the following extinct crocodylians: (1) Neogene and Quaternary gavialids in Asia (*Toyotamaphimeia machikanensis* from Japan [MOU F00001], *Penghusuchus pani* from Taiwan [NMNS 005645], and an indeterminate gavialine from Myanmar [MZKB F1280]); (2) Eocene crocodylians from the Bridger Formation of Wyoming (“*Crocodylus*” *affinis* [YPM 258], “*Crocodylus*” *grinnelli* [YPM 1344], *Boverisuchus vorax* [AMNH 29993; USNM 12957], and *Borealosuchus wilsoni* [USNM 12990]); (3) a giant alligatoroid *Deinosuchus riograndensis* (AMNH 3073) from the Campanian of Texas. We also extended the application of the current method to (4) a dwarf noncrocodylian neosuchian *Pachycheilosuchus trinquei* (USNM 427794) from the Albian of Texas (Supplementary data, Table S2). *Pachycheilosuchus trinquei* was previously recovered as a close relative of atoposaurids (Rogers 2003; Bronzati et al. 2012) or hylaeochampsids (Buscalioni et al. 2011; Turner and Pritchard 2015). *Pachycheilosuchus trinquei* is a noncrocodylian neosuchian and may not share the same vertebral formula with extant crocodylians; therefore, the body length estimates of *P. trinquei* should be interpreted with caution.

## Results

**Fig. 4:**
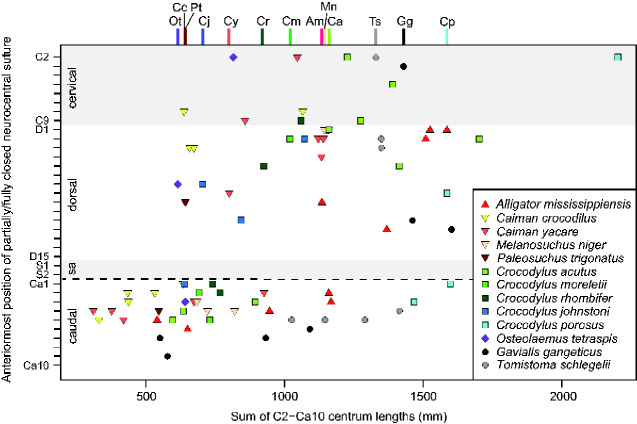
Anteriormost positions of partially/fully closed NC sutures in 79 individuals from 13 species. Colored bars on top of the graph represent the smallest osteologically mature individuals (individuals showing partially/fully closed precaudal NC sutures) for 13 species. Am, *A. mississippiensis*; Ca, *C. acutus*; Cc, *C. crocodilus*; Cj, *C. johnstoni*; Cm, *C. moreletii*; Cp, *C. porosus*; Cr, *C. rhombifer*; Cy, *C. yacare*; Gg, *G. gangeticus*; Mn, *M. niger*; Ot, *O. tetraspis*; Pt, *P. trigonatus*; Ts, *T. schlegelii*.

CLs of the axis to 10th caudal vertebra and their states of NC suture closure were documented for 95 individuals from 18 extant species (Fig. 4; Supplementary data, Table S1).

For the estimations of body lengths in extinct crocodylians, the axis to 10th caudal vertebra was divided into 10 morphologically distinct regions (Fig. 3). The mean differences (%) between the minimum and average CLs and the maximum and average CLs in seven vertebral regions with multiple vertebrae are shown in Table 1. CL measurements are expected to differ from the averages by 2.0–5.8% in each vertebral region. For example, the mean difference between the length of the shortest 3rd dorsal centrum and the average CL within 3–10th dorsal vertebrae in 95 individuals is 5.7% (Table 1). A summary of the log–log regressions of SVLs and TLs against the average CLs within each vertebral region and the sum of CLs in a series of vertebrae for 30 individuals from 10 species with SVL and/or TL records is shown in Table 2. *R*^2^ values were >0.95 for all regressions, indicating the strong correlations between the body lengths (SVLs and TLs) and the CLs.

**Table 1:** Mean differences (%) between the minimum/maximum and average CLs in seven vertebral regions among 95 individuals from 18 species

Mean difference (%) from the average CL	C2	C3–7 ave	C8–D2 ave	D3–10 ave	D11–14 ave	D15	S1–2 ave	Ca1	Ca2–4 ave	Ca5–10 ave
The minimum CL	–	4.5	5.0	5.7	4.3	–	3.0	–	5.8	2.5
The maximum CL	–	3.8	5.3	5.6	3.3	–	3.0	–	5.2	2.0

ave, average; C, cervical; Ca, caudal; D, dorsal; S, sacral.

**Table 2:** Regressions of SVLs and TLs against the average CLs within each vertebral region and the sum of CLs in a series of vertebrae using the individuals with SVL and TL records (*n* = 30)

Independent variable (x)	*R* ^2^	*P*-value	Elevation	Slope	Slope 95% Cl	
log(SVL) on log(x)						
C2	0.972	<0.001	1.472	0.999	0.936–1.066	
C3–7ave	0.980	<0.001	1.710	0.961	0.909–1.015	
C8–D2ave	0.985	<0.001	1.735	0.953	0.909–0.999	
D3–10ave	0.981	<0.001	1.626	0.990	0.939–1.043	
D11–14ave	0.980	<0.001	1.640	0.974	0.922–1.029	
D15	0.978	<0.001	1.717	0.956	0.903–1.011	
S1–2ave	0.978	<0.001	1.630	0.950	0.898–1.006	
Ca1	0.968	<0.001	1.681	1.001	0.934–1.072	
Ca2–4ave	0.975	<0.001	1.591	1.011	0.951–1.075	
Ca5–10ave	0.974	<0.001	1.589	0.984	0.924–1.047	
C2–9sum	0.981	<0.001	0.810	0.966	0.916–1.018	
D1–15sum	0.983	<0.001	0.495	0.980	0.931–1.031	
C2–D15sum	0.984	<0.001	0.329	0.976	0.929–1.025	
log(TL) on log(x)						
C2	0.955	<0.001	1.751	1.002	0.923–1.087	
C3–7ave	0.962	<0.001	1.990	0.963	0.894–1.039	
C8–D2ave	0.970	<0.001	2.015	0.956	0.894–1.022	
D3–10ave	0.972	<0.001	1.906	0.993	0.930–1.059	
D11–14ave	0.977	<0.001	1.920	0.976	0.920–1.036	
D15	0.971	<0.001	1.997	0.958	0.897–1.024	
S1–2ave	0.972	<0.001	1.910	0.953	0.893–1.017	
Ca1	0.953	<0.001	1.961	1.003	0.923–1.091	
Ca2–4ave	0.960	<0.001	1.871	1.014	0.939–1.095	
Ca5–10ave	0.959	<0.001	1.868	0.987	0.912–1.067	
C2–9sum	0.964	<0.001	1.088	0.969	0.900–1.042	
D1–15sum	0.974	<0.001	0.771	0.982	0.923–1.045	
C2–D15sum	0.973	<0.001	0.605	0.978	0.918–1.043	

ave, average; SVL, TLs, and CLs are in mm.

**Fig. 5:**
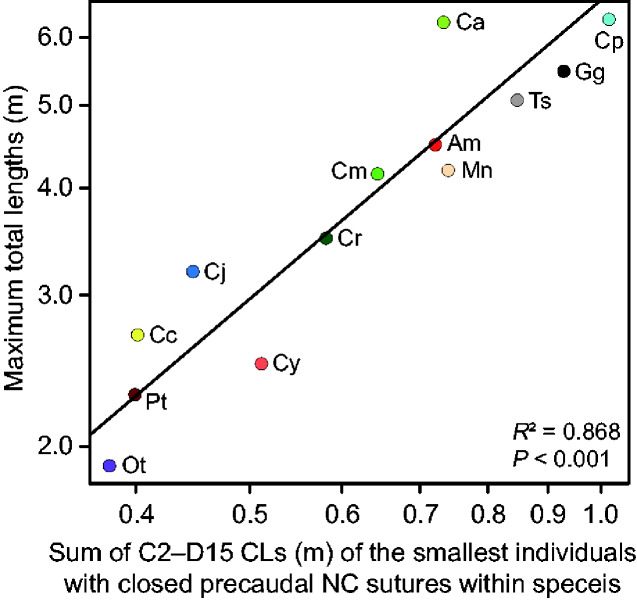
A relation between the maximum TLs and the sum of C2–D15 CLs of the smallest individuals with closed precaudal NC sutures for 13 species. Am, *A. mississippiensis*; Ca, *C. acutus*; Cc, *C. crocodilus*; Cj, *C. johnstoni*; Cm, *C. moreletii*; Cp, *C. porosus*; Cr, *C. rhombifer*; Cy, *C. yacare*; Gg, *G. gangeticus*; Mn, *M. niger*; Ot, *O. tetraspis*; Pt, *P. trigonatus*; Ts, *T. schlegelii*.

SVLs and TLs of the smallest osteologically mature individuals (individuals showing partially/fully closed precaudal NC sutures), male and female mature TLs, and the maximum TLs for 13 extant species are shown in Table 3. Missing SVLs (mm) and TLs (mm) of the smallest osteologically mature individuals of 13 extant species were estimated from the following equations using 30 individuals with the body length records: SVL = 1.329(CL_C2Ca10sum_)^0.980^; TL = 2.504(CL_C2Ca10sum_)^0.983^, where CL_C2–Ca10sum_ stands for the sum of the CLs of the axis to 10th caudal vertebra. Three species (*Melanosuchus niger*, *O. tetraspis*, and *Tomistoma schlegelii*) did not have male mature TL records, thus those values were estimated from female mature TLs using the average ratio of male and female mature TLs in 10 other species (Table 3). The smallest osteologically mature TLs were equal or larger than both male and female sexually mature sizes in 10 species, and between male and female sexually mature sizes in three species (*Paleosuchus trigonatus*, *Crocodylus acutus*, and *Crocodylus johnstoni*). On average, the smallest osteologically mature TLs were 1.09 and 1.27 times larger than males and female mature sizes, respectively. A summary of the log–log regressions of the species maximum TLs against the CLs (average CLs within each vertebral region and sum of the CLs in a series of vertebrae) of the smallest osteologically mature individuals for 13 species is shown in Table 4. *R*^2^ values were >0.83 for all regressions, indicating strong correlations between the species maximum TLs and the CLs in extant crocodylians (Fig. 5 and Table 4).

**Table 3:** SVLs and TLs of the smallest osteologically mature individuals (individuals showing partially/fully closed precaudal NC sutures), male and female sexually mature TLs, and the maximum TLs for 13 extant species

Species	Specimen #	Smallest osteologically mature SVL (m)	Smallest osteologically mature TL (m)	Species male mature TL (m)	Species female mature TL (m)	Species max. TL (m)	Literature for sexually mature male/female TLs	Literature for the maximum TLs
*Alligator mississippiensis*	UF 67824	1.31	2.53	1.83	1.83	4.50	Joanen and McNease 1975, 1980; Vliet 2020, 178	Brunell et al. 2015; Vliet 2020, 210
*Caiman crocodilus*	USNM 313860	0.75	1.43	1.43	1.30	2.70	Staton and Dixon 1977; Thorbjarnarson 1994	Velasco and Ayarzaguena 2010
*Caiman yacare*	UF 121245	0.91	1.71	1.56	1.30	2.50	Crawshaw 1987	Medem 1983, 171
*Melanosuchus niger*	UF 72914	1.29	2.59	2.38^a^	2.05	4.20	Herron 1991; Thorbjarnarson 2010	Medem 1981, 107; Thorbjarnarson 2010
*Paleosuchus trigonatus*	USNM 213705	0.66	1.15	1.31	1.10	2.30	Magnusson and Lima 1991 ^b^	Medem 1981, 112
*Crocodylus acutus*	UF 63930	1.40	2.66	2.82	2.20	6.25	Thorbjarnarson 1989 ^c^	Alvarez del Toro 1974; Pérez-Higareda et al. 1991
*Crocodylus johnstoni*	NTM JA076	0.82	1.58	1.68	1.40	3.20	Webb et al. 1983	Webb and Manolis 1989, 95
*Crocodylus moreletii*	UF 54813	1.18	2.27	1.50	1.50	4.16	Platt et al. 2008, 2009	Pérez-Higareda et al. 1991
*Crocodylus porosus*	FMNH 22026	1.82	3.51	3.35	2.30	6.30	Webb and Manolis 1989, 64^d^	Britton et al. 2012
*Crocodylus rhombifer*	AMNH 57773	1.07	2.06	1.97	1.91	3.50	Targarona et al. 2010	Targarona et al. 2010 ^e^
*Osteolaemus tetraspis*	UF 33749	0.72	1.38	1.02^a^	0.88	1.90	Shirley et al. 2017	Eaton 2010; Grigg and Kirshner 2015
*Gavialis gangeticus*	AMNH 110145	1.64	3.16	3.00	2.60	5.48	Whitaker and Basu 1982; Acharjyo et al. 1991; Stevenson and Whitaker 2010	Wermuth 1964; Grigg and Kirshner 2015
*Tomistoma schlegelii*	UF 84888	1.56	3.00	2.90^a^	2.50	5.07	Bezuijen et al. 2010	Wermuth 1964; Bezuijen et al. 2010^f^

^a^ Male sexually mature TLs for *M. niger*, *O. tetraspis*, and *T. schlegelii* were estimated from female mature TLs using the average ratio of male and female mature TLs in 10 other species.

^b^ Asymptotic SVL was considered as sexually matured size in Magnusson and Lima (1991). SVL was converted to TL using the dataset of Lemaire et al. (2018).

^c^ Sexually mature TLs of *C. acutus* vary across regions (e.g., Mazzotti 1983: Florida; Platt et al. 2011: Belize). We used data from Etang Saumâtre, Haiti (Thorbjarnarson 1989) because both male and female sexually mature sizes are available there.

^d^ Captive *C. porosus* matures early (male 3.0 m; female 2.1 m: Webb and Manolis 1989, 64).

^e^ A possible 5.03 m (16.5 ft) *C. rhombifer* (Gundlach 1880, 23) was not adopted here due to the large discrepancy from other accounts.

^f^ Estimated from 839 mm skull (BMNH 94.2.21.1) using the dataset of *T. schlegelii* (Wermuth 1964).

**Table 4:** Regressions of the species maximum TLs against the CLs (average CLs within each vertebral region and sum of the CLs in a series of vertebrae) of the smallest osteologically mature individuals for 13 species

Independent variable (x)	*R* ^2^	*P*-value	Elevation	Slope	Slope 95% CI	
log(max. TL) on log(x)						
C2	0.835	<0.001	1.649	1.220	0.935–1.592	
C3–7ave	0.886	<0.001	1.879	1.215	0.972–1.517	
C8–D2ave	0.878	<0.001	1.937	1.190	0.946–1.497	
D3–10ave	0.847	<0.001	1.991	1.103	0.853–1.427	
D11–14ave	0.863	<0.001	1.936	1.134	0.889–1.446	
D15	0.848	<0.001	1.859	1.236	0.957–1.597	
S1–2	0.832	<0.001	1.726	1.243	0.951–1.626	
Ca1	0.870	<0.001	1.853	1.255	0.990–1.590	
Ca2–4	0.880	<0.001	1.761	1.255	1.000–1.576	
Ca5–10	0.860	<0.001	1.678	1.275	0.997–1.631	
C2–9sum	0.882	<0.001	0.749	1.218	0.972–1.527	
D1–15sum	0.858	<0.001	0.633	1.129	0.881–1.446	
C2–D15sum	0.868	<0.001	0.344	1.159	0.913–1.472	

ave, average; TLs and CLs are in mm.

**Fig. 6:**
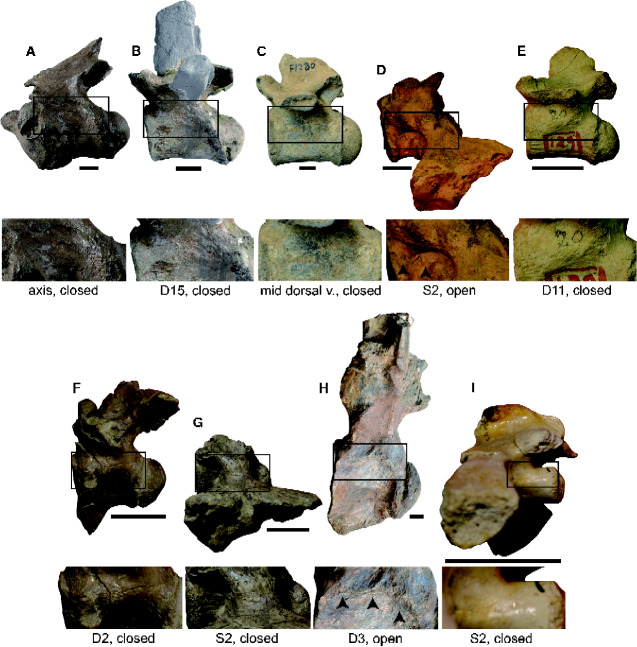
States of neurocentral suture closure in selected vertebrae of (**A**) *Toyotamaphimeia machikanensis* (MOU F00001), (**B**) *Penghusuchus pani* (NMNS 005645), (**C**) Gavialinae sp. indet. (MZKB F1280), (**D**) “*Crocodylus*” *affinis* (YPM 258), (**E**) “*Crocodylus*” *grinnelli* (YPM 1344), (**F**) *Boverisuchus vorax* (USNM 12957), (**G**) *Borealosuchus wilsoni* (USNM 12990), (**H**) *Deinosuchus riograndensis* (AMNH 3073), and (**I**) *Pachycheilosuchus trinquei* (USNM 427794). D, dorsal; S, sacral. Enclosed areas are enlarged below for each vertebra. Arrows represent open sutures. Scale bars equal 2 cm.

The vertebrae-based estimations of absolute SVLs and TLs and the upper and lower limits of the maximum TLs were applied to nine extinct crocodylians and an extinct noncrocodylian neosuchian (Table 5). Three post-Paleogene gavialids from Asia are osteologically mature (showing closed NC sutures in precaudal vertebrae), indicating those individuals reached sexual maturity (Fig. 6A–C). All three taxa are large with the absolute TLs of 4.49–7.01 m, and the maximum TLs of <9.00–14.02 m. Among Eocene crocodylians from the Bridger Formation of Wyoming, *C. grinnelli*, *B. vorax*, and *B. wilsoni* are sexually mature (Fig. 6E–G), while *C. affinis* (Fig. 6D) is osteologically immature and its sexual maturity is uncertain. The absolute and the maximum TLs of *C. affinis* are 3.87 m and >7.54 m, respectively. *Crocodylus grinnelli* and *B. vorax* are smaller species with their absolute TLs of 2.09–2.21 m and the maximum TLs of <3.63–3.88 m. *Borealosuchus wilsoni* is a relatively large species with its TL of 3.19 m and the maximum TL of <5.99 m. Sexual maturity of an alligatoroid *D. riograndensis* is unknown because the only available precaudal vertebra (possibly a third dorsal vertebra) shows an open NC suture (Fig. 6H). A dwarf neosuchian *P. trinquei* is sexually mature (Fig. 6I). The absolute TLs of *D. riograndensis* and *P. trinquei* are 7.73 and 1.15 m, respectively, and the maximum TL of the latter is <1.69 m.

**Table 5:** Vertebrae-based estimations of absolute SVLs and TLs and the maximum TLs in extinct crocodyliforms

Taxon	Specimen #	Formation and age	Anteriormost vertebra with closed NC suture	CL(s) used for SVL and TL estimation	Vertebrae-based SVL (m)	Vertebrae-based TL (m)	Vertebrae-based max. TL (m)	>Skull/mandible-based TL (m)	Femur length-based TL (m)	Literature for previous TL estimates based on skull/mandible and femur lengths
*Toyotamaphimeia machikanensis*	MOU F00001	Upper Osaka Group, Osaka, Japan (Middle Pleistocene)	C2	C2–D15sum	3.28 (3.12–3.44)	6.32 (5.91–6.74)	<13.50 (10.67–19.02)	6.28–7.26	–	Estimated using datasets of *G. gangeticus* and *T. schlegelii* (Wermuth 1964; Sereno et al. 2001)
*Penghusuchus pani*	NMNS 005645	Yuwentao Formation, Penghu Island, Taiwan (Miocene)	C2	C2–D15sum	2.33 (2.26–2.41)	4.49 (4.27–4.68)	<9.00 (7.60–11.64)	–	–	–
Gavialinae sp. indet.	MZKB F1280	Irrawaddy Formation, central Myanmar (late middle Miocene–Early Pleistocene)	Mid dorsal vertebra	D3–10ave	3.63 (3.73–4.28)	7.01 (6.48–7.56)	<14.02 (10.94–21.93)	–	–	–
“*Crocodylus*” *affinis*	YPM 258	Bridger Formation, Wyoming (early to middle Eocene)	Ca1	C2–D15sum	2.01 (1.95–2.07)	3.87 (3.71–4.00)	>7.54 (6.59–9.70)	3.38–3.44	3.24	Farlow et al. 2005; Mannion et al. 2019
“*Crocodylus*” *grinnelli*	YPM 1344	Bridger Formation, Wyoming (early to middle Eocene)	D5	C2–D15sum	1.09 (1.06–1.11)	2.09 (2.02–2.15)	<3.63 (3.36–3.99)	–	–	–
*Boverisuchus vorax*	USNM 12957	Bridger Formation, Wyoming (early to middle Eocene)	C8	C2–D15sum	1.15 (1.13–1.17)	2.21 (2.15–2.27)	<3.88 (3.60–4.21)	2.98–3.01	3.57	Hurlburt et al. 2003; Farlow et al. 2005; Mannion et al. 2019
*Boverisuchus vorax*	AMNH 29993	Bridger Formation, Wyoming (early to middle Eocene)	C3	C2–D15sum	1.12 (1.10–1.15)	2.15 (2.09–2.21)	<3.77 (3.51–4.12)			
*Borealosuchus wilsoni*	USNM 12990	Bridger Formation, Wyoming (early to middle Eocene)	S1	C2–D15sum	1.66 (1.62–1.70)	3.19 (3.08–3.27)	<5.99 (5.48–7.03)	4.26	4.54	Farlow et al. 2005
*Deinosuchus riograndensis*	AMNH 3073	Aguja Formation, Texas (Campanian)	Anterior dorsal vertebra	D3–10ave	4.01 (3.75–4.28)	7.73 (7.10–8.34)	–	8.43–10.64	7.67	Erickson and Brochu 1999; Schwimmer 2002; Farlow et al. 2005
*Pachycheilosuchus trinquei*	USNM 427794	Glen Rose Formation, Texas (Albian)	S1	S1–2ave	0.60 (0.58–0.63)	1.15 (1.09–1.21)	<1.69 (1.38–2.13)	–	1.13	Farlow et al. 2005

ave, average; Ranges in parentheses represent 95% CIs of SVL, TL, and the upper and lower limits of the maximum TL estimates. Previous TL estimates based on skull/mandible and femur lengths are shown for comparisons.

## Discussion

### Applicability and limitations of vertebrae-based body length estimations

This study is the first to provide vertebrae-based estimations of body lengths in crocodylians. Regional vertebral numbers are remarkably conservative among crocodylians (Reese 1915; Mook 1921b; Hoffstetter and Gasc 1969; Iijima and Kubo 2019), enabling the body length estimation using a single vertebra or a series of vertebrae. Because vertebrae have a high preservation potential due to their abundance, the current vertebrae-based method can complement the previous skull- and limb-based body length estimations (e.g., Wermuth 1964; Sereno et al. 2001; Farlow et al. 2005; O’Brien et al. 2019). Moreover, the maturity assessment using NC suture closure made it possible to reconstruct the species-specific body lengths (sexually mature and the maximum body lengths) in extinct crocodylians for the first time.

Comparison between the TLs of the smallest osteologically mature individuals (individuals showing partially/fully closed precaudal NC sutures) and male and female sexually mature TLs in 13 species showed that closed precaudal NC sutures can be used as an indicator of sexual maturity (Table 3). The smallest osteologically mature TLs were larger than male sexually mature TLs in 10 out of 13 species, and larger than female sexually mature TLs in all 13 species, suggesting that precaudal NC sutures generally start to close after sexual maturity in extant crocodylians (Table 3). The current observation across extant crocodylians is consistent with the previous studies of NC suture closure in crocodylians (Brochu 1992, 1996; Ikejiri 2012). Ikejiri (2012) examined 75 *A. mississippiensis* and demonstrated that all precaudal NC sutures remain open at sexually mature sizes in males and females (TL = 1.83 m; Joanen and McNease 1975, 1980; Vliet 2020). Brochu (1992, 1996) examined the timings of NC suture closure in two large species (*A. mississippiensis* and *C. acutus*) and two dwarf species (*Alligator sinensis* and *O. tetraspis*), showing that precaudal NC sutures close at different sizes in large and dwarf species. He also noted slightly accelerated suture closure in *C. acutus* compared to *A. mississippiensis* (Brochu 1992, 1996), which is congruent with the current results (Fig. 5 and Table 3).

Although a closed precaudal NC suture seems to be a sound sexual maturity indicator for both males and females, there are several limitations to the current dataset. First, poor sampling might underestimate the smallest osteologically mature TLs in several species (*M. niger*, *C. acutus*, *Crocodylus moreletii*, and *G. gangeticus*: Fig. 4; Supplementary data, Table S1). Second, intraspecific variation in the timings of NC suture closure was not assessed due to the small sample size and sparsity of sex data. In extant crocodylians, males grow larger than females (Webb and Manolis 1989; Vliet 2020), and male sexual maturity sizes are ∼17% larger than those of females on average (Table 3). The disparity of male and female sexually mature sizes is most pronounced in *Crocodylus porosus*, where the male maturity size is 46% larger than that in females (Webb and Manolis 1989). Moreover, sexual maturity can be reached at smaller sizes in captive environments (Webb and Manolis 1989; Platt et al. 2010), which would be the case in our captive specimens (28 individuals). Furthermore, sexual maturity sizes may differ across populations and geographical regions (Webb et al. 1983; Thorbjarnarson 1989), which was not taken into account here. The effects of these factors (sex, wild-caught/captive-raised, population, and geography) on timings of NC suture closure need to be investigated with a larger dataset in the future.

The current vertebrae-based method provides insights into the maximum sizes of extinct crocodylians. In extant crocodylians, the species maximum TLs are strongly correlated with the CLs of the smallest osteologically mature individuals (*R*^2^ > 0.83) (Fig. 5 and Table 4). Therefore, combined with the state of NC closure (closed precaudal vertebrae or open caudal vertebrae), the upper or lower limit of the species maximum TL can be determined from the CLs. Vertebrae of multiple individuals from a single species can narrow the range of the maximum TL; the smallest closed precaudal vertebra and the largest open caudal vertebra constrain the upper and lower limit, respectively. Meanwhile, if the maximum TL in one individual is smaller than the absolute TL in the other individual, those can be distinguished as different species. Limitations to the current dataset are the accuracy of the maximum TL records in extant species and the difficulty in estimating the exact maximum TLs of extinct crocodylians, as well as the poor sampling and intraspecific variation discussed above. We adopted only the measured maximum TL records, while unconfirmed reports and eyewitnesses that could represent the maximum TLs were not considered (Webb and Manolis 1989). Besides, the ongoing search for larger individuals of extant species may break the current maximum TL records in the future (Whitaker and Whitaker 2008). Estimations of exact maximum TLs are challenging because precaudal NC sutures close rapidly toward the axis once the sacral vertebrae start to close (Brochu 1996; Ikejiri 2012). Consequently, there is a large intraspecific variation in the anteriormost positions of closed vertebrae among similar-sized individuals (Brochu 1996; Ikejiri 2012).

### Body length estimations in extinct crocodyliforms

Among three Asian post-Paleogene gavialids (Table 5), *T. machikanensis* preserves its skull and allows comparison of vertebrae- and skull-based TL estimates. For the TL estimations from skull lengths, the measurements of two extant gavialids (*G. gangeticus* and *T. schlegelii*: Wermuth 1964; Sereno et al. 2001) were used for RMA regressions given the uncertain phylogenetic position of *T. machikanensis* (Iijima and Kobayashi 2019). The vertebrae-based TL estimate of *T. machikanensis* (6.32 m) is comparable to the skull-based TL estimates using the extant gavialids (6.28–7.26 m; Table 5), confirming the utility of the vertebrae-based method. In *T. machikanensis* and *P. pani*, NC sutures are partially/fully closed in almost all precaudal vertebrae, thus these individuals reached sexual maturity much earlier, and their maximum TL estimates are of little utility. All three Asian gavialids examined here are large taxa, with absolute TL estimates of 4.49–7.01 m (Table 5). Because extant gavialids (*G. gangeticus* and *T. schlegelii*) are also large taxa (maximum TLs > 5 m; Table 3), and the middle Miocene gavialid *Rhamphosuchus crassidens* was estimated as 8–11 m TL (Head 2001), Asian post-Paleogene gavialids may form a large-bodied group. Prior to the Neogene, several possible gavialids are known from the Eocene of Central, South, Southeast, and East Asia (Yeh 1958; Sahni and Mishra 1975; Efimov 1982, 1988; Böhme et al. 2011; Shan et al. 2017; Martin et al. 2019). Most of these materials are fragmentary, while *Maomingosuchus petrolica* is represented by hundreds of skeletons from the late Eocene of Guangdong Province, China (Yeh 1958; Shan et al. 2017). The largest skull is 503 mm in length (Shan et al. 2017), which gives the TL estimates of 2.98–2.99 m based on the extant gavialid measurements, suggesting that *M. petrolica* is a medium-sized crocodylian. The phylogenetic relationship of gavialids needs to be resolved to reconstruct the body size evolution in Asian gavialids.

The middle–late Eocene Bridger Formation and the stratigraphically correlated lower Washakie Formation of Wyoming (Roehler 1973; Murphey and Evanoff 2011) hosted a wide array of crocodylians including *B. wilsoni* (Mook 1959; Brochu 1997a), a planoclaniid *B. vorax* (Troxell 1925a; Langston 1975; Brochu 2013), alligatorines *Allognathosuchus polyodon* and *Procaimanoidea kayi* (Cope 1873; Mook 1921c, 1941, 1961; Brochu 2004), and genus *Crocodylus* (Marsh 1871; Cope 1882; Mook 1921d, 1933b; Troxell 1925a; Gilmore 1946; Steel 1973; Norell and Storrs 1989; Brochu 1997b, 2000). Examinations of nonalligatoroid taxa revealed the body size diversity within the formations. *Crocodylus affinis* (YPM 258) is a large species (TL = 3.87 m, maximum TL > 7.54 m), and *C. grinnelli* (YPM 1344) is a medium-sized species (TL = 2.09 m; maximum TL < 3.63 m). *Crocodylus affinis* (YPM 258) is osteologically immature at 3.87 m TL, which is larger than sexual maturity sizes of all extant crocodylians (Table 3). The upper limit of the maximum TL estimate of *C. grinnelli* (YPM 1344) is below the absolute TL estimate of *C. affinis* (YPM 258) although their 95% CIs slightly overlap (Table 5), thus these specimens probably belong to different species. Our result contradicts the previous conclusion that *C. grinnelli* is a junior synonym of *C. affinis* (Norell and Storrs 1989; Brochu 1997b). Since YPM 258 is only represented by postcrania and tentatively referred to as *C. affinis* (Norell and Storrs 1989), additional materials with associated cranial and axial elements need to be examined to confirm the body length difference between *C. affinis* and *C. grinnelli*. Previous TL estimates of *C. affinis* (USNM 12719) were 3.00–3.44 m based on the skull and femur lengths and the mounted skeleton (Gilmore 1946; Farlow et al. 2005; Mannion et al. 2019; Table 5). If YPM 258 and USNM 12719 are conspecific, the latter should show open precaudal NC sutures. A planocraniid *B. vorax* is a medium-sized species (TL = 2.15–2.21 m; maximum TL < 3.77–3.88 m) comparable to the size of *C. grinelli*, while *B. wilsoni* is a large species (TL = 3.19 m; maximum TL < 5.99 m). Because the anteriormost position of the closed NC suture is the first sacral vertebra in *B. wilsoni* (USNM 12990), its maximum TL would be close to 6 m. Previous skull- and femur length-based TL estimates for *B. vorax* (2.98–3.57 m) and *B. wilsoni* (4.26–4.54 m) are larger than their vertebrae-based TL estimates, but still smaller than their maximum TL estimates (Farlow et al. 2005; Mannion et al. 2019; Table 5). Diverse body lengths in non-alligatoroid crocodylians in the Bridger and Washakie formations, combined with their morphological specializations (e.g., Langston 1975; Rossmann 2000), might facilitate the habitat partitioning among the sympatric species (Stout 2012).

An alligatoroid *Deinosuchus* from the Campanian of North America and a noncrocodylian neosuchian *P. trinquei* from the Albian of Texas are iconic giant and dwarf crocodyliforms, respectively, and their body sizes have long been debated (Colbert and Bird 1954; Brochu 1996; Erickson and Brochu 1999; Schwimmer 2002; Rogers 2003; Farlow et al. 2005). The TL estimate of *D. riograndensis* (AMNH 3073) based on a possible third dorsal vertebra is 7.73 m (Table 5). However, the third dorsal vertebra is the shortest in the anterior–mid dorsal region (3rd to 10th dorsal vertebrae), of which the average CLs in extant crocodylians were used for the TL estimation. Therefore, the TL estimate can be up to 5.7% larger and be 8.17 m (Table 1). An open NC suture in the third dorsal vertebra of *D. riograndensis* (AMNH 3073) prevents the sexual maturity assessment. The TL of the same material was previously estimated as 15.24 m (Colbert and Bird 1954), which is now regarded as an overestimate (Schwimmer 2002). *Deinosuchus* vertebrae larger than AMNH 3073 were known from the Judith River Formation of Montana (CM 963: Holland 1909), from which 12.0 m TL was roughly deduced in Schwimmer (2002). States of NC suture closure in CM 963 are hardly determined from the published drawings and figures (Holland 1909; Cossette and Brochu 2020). TLs of other *Deinosuchus* materials from Texas were estimated as 7.67 m from the femur length (TMM 43632-1; Farlow et al. 2005), 8.43–9.10 m (Erickson and Brochu 1999) and 11 m (Sues 1989) from mandibular remains, and 9.8 m (Schwimmer 2002) and 10.64 m (TMM 43632-1; Farlow et al. 2005) from skull lengths (Table 5). Additional vertebral remains are required to get a better understanding of the sexually mature and the maximum sizes of *Deinosuchus*.

*Pachycheilosuchus trinquei* is a dwarf species (TLs = 1.15 m, maximum TL < 1.69 m); it is osteologically mature and probably smaller than all extant crocodylians (Table 3). Previous TL estimates of *P. trinquei* were 0.64–0.80 m from a dentary and the composite reconstruction, and 1.13 m from the femur length (Farlow et al. 2005; Table 5). Examination of the state of NC suture closure in smaller precaudal vertebrae (Rogers 2003) may further constrain the upper limit of the maximum TL. Discussion of the body size dwarfism on the lineage of *P. tringuei* requires the establishment of its phylogenetic position.

**Fig. 7:**
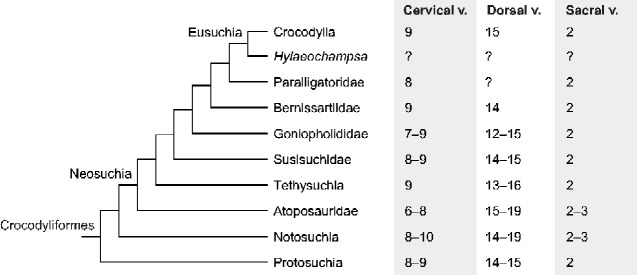
Numbers of cervical, dorsal, and sacral vertebrae in crocodyliforms. The phylogenetic relationships are based on Wilberg (2015) and Wilberg et al. (2019), supplemented by Turner (2015) and Tennant et al. (2016). Note that phylogenetic positions of many clades are still under debate (Puértolas-Pascual et al. 2020). Literature for vertebral counts of each clade: Paralligatoridae (Wu, Cheng, et al. 2001; Pol et al. 2009); Bernissartiidae (Norell and Clark 1990); Goniopholididae (Wu et al. 1996; Lauprasert et al. 2007; Erickson 2016; Martin et al. 2016; GMNH-PV0000229); Susisuchidae (Salisbury et al. 2003, 2006; Figueiredo et al. 2011); Tethysuchia (Troxell 1925b; Storrs 1986; Langston 1995; Wu, Russell, et al. 2001; Jouve and Schwarz 2004; Jouve et al. 2006; Schwarz et al. 2006); Atoposauridae (Wellnhofer 1971; Buscalioni and Sanz 1990; Michard et al. 1990; Storrs and Efimov 2000; Tennant et al. 2016); Notosuchia (Pol 2005; Turner 2006; Fiorelli and Calvo 2008; Sereno and Larsson 2009; Georgi and Krause 2010; Nascimento and Zaher 2010; O’Connor et al. 2010; Nobre and de Souza Carvalho 2013; Leardi et al. 2015; Martinelli et al. 2018); Protosuchia (Colbert and Mook 1951; Li 1985; Wu et al. 1994; Peng 1996; Osmólska et al. 1997; Pol and Norell 2004; Peng and Shu 2005). See Supplementary data, Table S3 for vertebral counts for each species.

The application of vertebrae-based estimations of absolute and species-specific body lengths in more inclusive groups (e.g., Neosuchia and Crocodyliformes) requires the following premises: (1) caudal to cranial NC suture closure sequence; (2) similar timings of NC suture closure (i.e., closure in precaudal vertebrae after sexual maturity); and (3) similar regional vertebral numbers and body proportions as compared to crown-group crocodylians. Caudal to cranial suture closure sequence is most likely shared among crocodyliforms and even among pseudosuchians, because anteriorly open and posteriorly closed vertebrae are found in non-crocodyliform pseudosuchians including an erpetosuchid *Parringtonia* (Nesbitt and Butler 2013), aetosaurians *Aetosauroides*, *Desmatosuchus*, and *Typothorax* (Case 1922; Martz 2002; Irmis 2007; Taborda et al. 2015), a basal crocodylomorph *Terrestrisuchus* (Irmis et al. 2013), metriorhynchoids *Pelagosaurus*, *Cricosaurus*, and *Tyrannoneustes* (Delfino and Dal Sasso 2006; Herrera et al. 2013; Young et al. 2013), and crocodyliforms such as a dyrosaurid *Guarinisuchus* (Barbosa et al. 2008). A comparison of relative timings of NC suture closure is challenging. A possible case of late suture closure was reported in *Notosuchus*, where all individuals, including the one with a very large skull, show open sutures in presacral vertebrae (Pol 2005). Whether all those individuals are relatively young (e.g., sexually immature individuals) is uncertain. Numbers of presacral vertebrae vary in noncrocodylian Neosuchia (Paralligatoridae, Bernissartiidae, Goniopholididae, Susisuchidae, Tethysuchia, and Atoposauridae), Notosuchia, and Protosuchia (*Protosuchus*, *Gobiosuchus*, *Zaraasuchus*, *Edentosuchus*, *Shantungosuchus*, *Sichuanosuchus*, and *Hsisosuchus*: Wilberg et al. 2019) (Fig. 7; see Supplementary data, Table S3 for vertebral counts for each species). Generally, there are 7–9 cervical, 13–17 dorsal, and 2–3 sacral vertebrae in crocodyliforms, and only a few taxa deviate from the range: possibly 6 cervical vertebrae in *Atoposaurus jourdani* (Lortet 1892; Tennant et al. 2016); 12 dorsal vertebrae in *Goniopholis* aff. *G. lucasii* (Erickson 2016); 19 dorsal vertebrae in *Brillanceausuchus babouriensis* (Michard et al. 1990; Tennant et al. 2016); 10 cervical and 19 dorsal vertebrae in *Notosuchus terrestris* (Pol 2005; Fiorelli and Calvo 2008). Additionally, Thalattosuchia, a group of aquatic noncrocodyliform crocodylomorphs (Wilberg 2015) slightly deviates from the precaudal vertebral formula of crocodyliforms (6–9 cervical, 15–19 dorsal, and 2–3 sacral vertebrae: Pierce and Benton 2006; Young et al. 2010; Herrera et al. 2013; Sachs et al. 2019; Johnson et al. 2020). Meanwhile, relative tail lengths in some notosuchian crocodyliforms are significantly shorter than those in crown-group crocodylians (e.g., *Simosuchus clarki* and *Caipirasuchus mineirus*: Georgi and Krause 2010; Martinelli et al. 2018). Given the small variation in the number of precaudal vertebrae in crocodyliforms, SVL estimation can be applied to crocodyliforms with caution, while TL estimation should best be applied to close relatives of crown-group crocodylians (e.g., Eusuchia or Neosuchia). Nonetheless, closed precaudal NC sutures can still be used as the sexual maturity indicator in extinct crocodyliforms on the assumption that timings of suture closure are similar as compared to crown-group crocodylians.

## Conclusion

Crown-group crocodylians share the same number of precaudal vertebrae, allowing the vertebrae-based estimations of their body lengths. Measurements of the CLs and assessments of states of NC suture closure provided insights into the absolute and species-specific body lengths (sexually mature and the maximum body lengths) in crocodylians. Most extant crocodylians reach sexual maturity before closure of precaudal NC sutures; therefore, closed sutures in precaudal vertebrae can be used as an indicator of sexual maturity. CLs of the smallest individuals with closed precaudal NC sutures within species were correlated with the species maximum TLs for 13 extant species; using this relationship, the upper or lower limit of the maximum TLs in extinct species can be derived from the smallest precaudal vertebrae with closed sutures and the largest caudal vertebrae with open sutures, respectively. The current method can potentially be applied to noncrocodylian crocodyliforms, although differences in the regional vertebral numbers, body proportions, and timings of NC suture closure need to be carefully considered.

## Supplementary Material

obaa042_Supplementary_Data
